# Efficient Fractionation of Lignin- and Ash-Rich Agricultural Residues Following Treatment With a Low-Cost Protic Ionic Liquid

**DOI:** 10.3389/fchem.2019.00246

**Published:** 2019-04-17

**Authors:** Clementine L. Chambon, Meng Chen, Paul S. Fennell, Jason P. Hallett

**Affiliations:** Laboratory of Sustainable Chemical Technology, Department of Chemical Engineering, Imperial College London, South Kensington Campus, London, United Kingdom

**Keywords:** ionic liquids, biorefining, lignocellulose, agroresidues, pretreatment, enzymatic hydrolysis, lignin

## Abstract

Agricultural residues from rice, wheat and sugarcane production are annually available at the gigaton-scale worldwide, particularly in Asia. Due to their high sugar content and ash compositions, their conversion to bioethanol is an attractive alternative to their present disposal by open-field burning and landfilling. In this work, we demonstrate application of the low-cost protic ionic liquid triethylammonium hydrogen sulfate ([TEA][HSO_4_]) for pretreatment of rice straw, rice husk, wheat straw and sugarcane bagasse. The feedstocks had high ash (up to 13 wt%) and lignin content (up to 28 wt%). Pretreatment effectiveness was examined at 150 and 170°C and an optimal pretreatment time was identified and characterized by glucose release following enzymatic saccharification (i.e., hydrolysis), biomass delignification observed by compositional analysis, and lignin recovery. The isolated lignin fractions were analyzed by 2D HSQC NMR to obtain insights into the structural changes occurring following ionic liquid pretreatment. After treatment at 170°C for 30–45 min, enzymatic hydrolysis of three agroresidues gave near-quantitative glucose yields approaching 90% while rice husk gave 73% yield. Glucose release from the pulps was enhanced by saccharifying wet pulps without an air-drying step to reduce hornification. According to pulp compositional analysis, up to 82% of lignin was removed from biomass during pretreatment, producing highly digestible cellulose-rich pulps. HSQC NMR of the extracted lignins showed that delignification proceeded *via* extensive cleavage of β-*O*-4′ aryl ether linkages which was accompanied by condensation reactions in the isolated lignins. The high saccharification yields obtained indicate excellent potential for valorization of low-cost agroresidues in large volumes, which is promising for commercialization of biofuels production using the ionoSolv pretreatment technology.

## Introduction

Agricultural residues (e.g., straws, husks, stalks, stovers and cobs) and residues originating from industrial processing (e.g., bagasse) are among the most abundant renewable resources on earth (nee' Nigam et al., 2009). These lignocellulosic materials are composed of up to 80 wt% polysaccharides, making them rich sources of energy (Reddy and Yang, 2005). Wheat, rice, maize and sugarcane production generate the vast majority of residues, with global resources of 2.8 Gtons of dry matter available annually (IEA Bioenergy, 2015) after discounting ~40% that must be left on the cropland to maintain the soil organic matter, carbon and nutrient balance (Scarlat et al., 2010). As such, agricultural crop residues represent a significant resource for bioenergy and biorefineries. Due to their abundance, high carbohydrate and ash content, agroresidues from rice, wheat and sugarcane production are most suitable for conversion to bioethanol. Kim and Dale (2004) have estimated that crop residues could be used to produce around 442 GL of bioethanol annually, of which 360 GL (>80%) could be produced from rice straw, wheat straw and bagasse alone. This amounts to over 14 times global bioethanol production (31 GL) (Kim and Dale, 2004). These feedstocks are most abundant in Asia (60%), which has the highest potential for bioethanol production from agricultural residues (Kim and Dale, 2004). As these are carbon-neutral resources that do not require additional land to produce, significant opportunities exist to reduce greenhouse gas emissions, increase domestic energy security and boost rural economies by displacing fossil fuels in the production of bioenergy and bio-based products.

Most agricultural residues are not optimally utilized at present, since their digestibility by animals and in established conversion processes is usually low (IEA Bioenergy, 2015). They are often considered waste materials and are disposed of by open-field burning, landfilling or incineration (Mehta and Pitt, 1976). Notably, open-field burning of rice, corn and wheat straws in South Asia and China has led to a pressing environmental crisis, releasing huge quantities of black carbon, the second-largest human emission causing climate change (Bond et al., 2013). Biomass burning also results in particulate and gaseous air pollution, with devastating consequences for public health and the environment (Shih et al., 2008). Accumulation of agricultural residues leads not only to the deterioration of the environment, but also to the loss of a potentially valuable feedstock which could be processed to yield a variety of fuels, chemicals and materials. If all rice straw that is currently burned were instead converted into bioethanol, it would become the single largest feedstock for lignocellulosic ethanol production (Satlewal et al., 2018). Despite their remarkable potential for conversion to bioethanol, these feedstocks are not presently used in commercial biofuels production processes due to low enzyme digestibility, linked to their high lignin content.

Pretreatment is an important tool for breakdown of the recalcitrant structures within agricultural residues prior to biochemical conversion. For bioethanol production, an effective and economical method is needed to separate polysaccharides from lignin, ash and other constituents in native biomass. However, the presence of high lignin and ash content in many agricultural feedstocks is problematic for conventional pretreatment processes, with sugar yields typically limited to 50–60% (Saha et al., 2005). Several important agricultural crops produce residues which are highly recalcitrant and also contain up to 30% ash by weight, notably rice straw and rice husk (Parikh et al., 2005). High ash concentrations are unfavorable for feedstock pre-processing and the extraction of cellulose. Silica, a major ash constituent, wears down machinery used for feedstock grinding (Miles et al., 1996). Ash can also react with acids (i.e., the acidic pretreatment medium itself, or added acid catalyst) during dilute acid pretreatment, hot water or steam explosion pretreatment, increasing the amount of chemicals required for processing (Huang et al., 2017). Dilute acid pretreatment is a leading pretreatment process suitable for commercialization, but it is unable to delignify biomass with >10 wt% lignin, as is the case for many agroresidues (Fu et al., 2010). Steam explosion and hydrothermal pretreatments are somewhat suited for more lignified materials; however, the pretreated materials are poorly converted during enzymatic hydrolysis, presenting maximum glucose yields of around 60% at high solid to liquid ratio (i.e. solids loading) of 1:2 g/g wt% (Sun and Cheng, 2002). Delignifying pretreatments, such as treatment with alkali or with organic or aqueous-organic solvents (“organosolv” processing), have shown some success in pretreating the most recalcitrant agroresidue, rice husk (Singh et al., 2011; Singh and Dhepe, 2016), as well as rice straw (Zhang and Cai, 2008; Amiri et al., 2014). However, these methods have their respective drawbacks, including high reagent costs, and are considered economically unviable for bioethanol production from lignocellulose (Sánchez and Cardona, 2008). While alkaline pretreatment can be highly effective at delignifying a wide range of herbaceous feedstocks, performance suffers under high solids loadings representative of industrial biorefining, where glucose yields were limited to ~50% (Cheng et al., 2010), thus high chemicals consumption is a major challenge for commercialization (Chen et al., 2013). In organosolv pretreatments, the organic solvents used are normally flammable and can generate explosive atmospheres, requiring strenuous safety precautions and leading to very high cost (Zhao et al., 2009). Generally, the use of agricultural residues is also hindered by their low bulk density and low energy density (Mani et al., 2004; Hoover et al., 2014) which require the development of high-efficiency pretreatment processes.

Ionic liquid biorefining has emerged as among the most promising technologies for large-scale economical conversion of agroresidues to bioethanol and fine chemicals (Yoon et al., 2011). Ionic liquids (ILs) are salts with low melting points that are thermally stable, non-volatile and non-flammable (Brandt et al., 2013). ILs are generally observed to have extremely low vapor pressures (Earle et al., 2006), avoiding environmental and safety hazards during handling. This also makes many ILs reusable and recyclable, offering a clean and safe alternative to conventional organic solvents (Brandt-Talbot et al., 2017). The recent surge of interest in ILs is due to their ability to decrystallize or dissolve cellulose (Swatloski et al., 2002). Cellulose-dissolving ILs have been reported to give high conversions with a variety of lignocellulose feedstocks (Li et al., 2010). Among these, the widely studied aprotic IL 1-ethyl-3-methylimidazolium acetate ([Emim][OAc]) has been shown to release over 90% of glucose from sugarcane bagasse at a moderate solids loading of 1:6.7 g/g (Fu et al., 2010). However, the high cost of alkylimidazolium ILs as well as their low thermal stability (Clough et al., 2013) and the requirement for dry conditions limits their ability to be economically recycled and reused in commercial processing (George et al., 2015). Therefore, a class of low-cost amine-based protic ILs was developed by Hallett et al. (Chen et al., 2014). Among these, alkylammonium hydrogen sulfate ILs are significantly more thermally stable than [Emim][OAc] and can be recycled by distillation to regenerate a concentrated IL solution and reused (Chen et al., 2014; George et al., 2015; Brandt-Talbot et al., 2017).

IonoSolv pretreatment is a lignocellulose fractionation technology that uses IL-water mixtures to extract lignin and hemicellulose from biomass, leaving behind a cellulose-rich pulp (Brandt et al., 2011). It uses thermally stable, recyclable and inexpensive protic ILs that are suitable for large-scale biomass processing (Chen et al., 2014; George et al., 2015; Brandt-Talbot et al., 2017). Protic ILs can be produced by mixing a Brønsted acid with a Brønsted base in a single step (Greaves and Drummond, 2015). The solvent of choice in this study is triethylammonium hydrogen sulfate [TEA][HSO_4_] that can be cheaply manufactured, with bulk cost as low as $1.24 kg^−1^ (Chen et al., 2014), at least 40 times cheaper than [Emim][OAc] (George et al., 2015). Brandt-Talbot et al. (2017) previously showed that [TEA][HSO_4_] is a promising candidate protic IL for use in economically viable pretreatment processes. The IL was successfully recovered and reused four times for pretreatment of *Miscanthus*, and saccharification yields for *Miscanthus* were not affected by IL recycling (Brandt-Talbot et al., 2017). It has also been applied to pretreat sugarcane bagasse under mild temperature conditions (120°C, 4 h), resulting in glucose yields of up to 65% (Chambon et al., 2018). Recently, Gschwend et al. (2018) demonstrated that process temperatures up to 180°C improved lignin extraction and delignification and reduced residence times from hours to minutes, suggesting that this IL could be similarly applied to highly lignified feedstocks (up to 30 wt%). A major limitation of our previous work is that pretreated pulps were air-dried before enzymatic saccharification, severely limiting glucose yields. Therefore, in an attempt to reflect industrial practice, in this w we have avoided the energy-intensive air-drying step and directly hydrolyzed wet pulps to observe ionoSolv process performance on suitable candidate agricultural feedstocks for the future biorefinery.

In this work, we investigated the suitability of lignin-dissolving IL [TEA][HSO_4_] for pretreatment of four agricultural residues, namely rice straw, rice husk, sugarcane bagasse and wheat straw. The four materials have high lignin (28 wt%) and ash content (up to 13 wt%) yet they are ideal feedstocks for biorefining as they are highly abundant, geographically diverse, low-cost and easily procured (Reddy and Yang, 2005). Here we apply ionoSolv processing at 150 and 170°C to establish optimal treatment times for cellulose valorization. Another novelty is the determination of the effect of pulp drying (pore collapse) on sugar yield. Successful pretreatment was characterized by high glucose release, delignification and lignin recovery, as gauged by enzymatic hydrolysis and compositional analysis of the recovered pulps. Subsequently, isolated lignin fractions were compared by 2D HSQC NMR to obtain insights into the structural changes occurring during ionoSolv pretreatment.

## Materials and Methods

Rice husk (*Oryza sativa*) was obtained from a rice mill in Bahraich district, Uttar Pradesh, India; rice straw was helpfully provided by the Institute of Chemical Technology, Mumbai, after harvesting in Tirunelveli district, Tamil Nadu, India; wheat straw (*Triticum aestivum*) was received from Glasgow, UK; depithed sugarcane bagasse (*Saccharum officinarum*) was kindly provided by Sugar Milling Research Institute, Durban, and originated from KwaZulu-Natal province, South Africa. All feedstocks were washed to remove adhering inorganic debris and air-dried at room temperature in order to prevent microbial degradation during shipping. Each feedstock was ground using a cutting mill (Retsch SM200, Germany) and then sieved using a vibratory shaker (Retsch AS200, Germany) to a particle size within 0.18–0.85 mm (US mesh scale −20/+80). The feedstocks were then stored air-dry in sealed plastic bags at ambient temperature. All chemical reagents were purchased from VWR International or Sigma-Aldrich and used as received. The Karl-Fischer titrator used to measure ionic liquid moisture content was a V20 volumetric Titrator (Mettler-Toledo). The analytical balance used in this study was a Sartorius CPA 1003S (±0.001 g).

### Ionic Liquid Synthesis

The ionic liquid triethylammonium hydrogen sulfate ([TEA][HSO_4_]) with an acid : base ratio of 1 : 1 (mol/mol) was synthesized according to a previously published protocol (Gschwend et al., 2018). The ionic liquid water content was adjusted to 20 wt% using a Karl-Fischer titrator (V20 volumetric titrator, Mettler-Toledo, USA) according to the standard operating procedure of our laboratory (Gschwend et al., 2016).

### IonoSolv Pretreatment

Pretreatments, determination of oven-dried weight and pulp and lignin yields were conducted following the standard operating procedure of our laboratory (Gschwend et al., 2016). The solvent used for all experiments was a mixture of [TEA][HSO_4_]:H_2_O (4:1 wt/wt), i.e., with a final water content of 20 wt%. Pretreatments were carried out in triplicates using 10 g of solvent and 1 g of biomass (on an oven-dried basis), corresponding to a biomass loading of 1:10 g/g. Pretreatments were performed at 150° and 170°C for a pretreatment time varying between 30 and 180 min depending on the feedstock and operating temperature. For experiments *with* air-drying of the pulp, the cellulose-rich material (pulp) was washed four times with ethanol (40 mL) and Soxhlet extracted for 24 h using ethanol in cellulose thimbles before being air-dried overnight. The pulp was weighed before being subjected to saccharification. The collected ethanol washes and Soxhlet extractives were combined and ethanol removed by evaporation using a rotavapor, generating an ethanol-free IL-water liquor. Lignin was precipitated by addition of water (40 mL) as anti-solvent to the liquor and lignin was isolated by washing with water before being dried under vacuum at 45°C.

For experiments *without* air-drying of the pulp, the procedure was followed unchanged until the Soxhlet extraction step. The pulp-containing thimbles were removed from the Soxhlet adapter after completion of the extraction and transferred to 50 mL Falcon tubes which were immediately filled with de-ionized water (40 mL). The pulp was left in the thimble inside the Falcon tube for at least an hour. The thimble was then taken out of the Falcon tube and the pulp transferred back to the Falcon tube using a spatula. The tubes were centrifuged (3,000 rpm or 2,000x*g*, 50 min) and the supernatant decanted; the washing step was repeated once more in de-ionized water. The wet pulp was weighed, its moisture content determined immediately and saccharification started the following day. Wet pulps were stored at 5°C.

### Saccharification Assay

The enzymatic hydrolysis assay was carried out according to an adapted procedure entitled “Low Solids Enzymatic Saccharification of Lignocellulosic Biomass” published by the NREL (Resch et al., 2015). Saccharification assays were performed on native biomass, air-dried pulps and wet pulps, each in triplicate, using Cellic® CTec2 enzymes (Novozymes, Denmark).

100 ± 5 mg (on an oven-dried weight basis) of air-dried or wet biomass was placed into a Sterilin tube and the weight recorded. Three blanks were run with 100 μL of purified water in order to correct for sugar residues present in the enzyme solutions. 9.9 mL solution consisting of 5 mL 1 M sodium citrate buffer at pH 4.8, 40 μL tetracycline antibiotic solution (10 mg/mL in 70% ethanol), 30 μL cycloheximide antibiotic solution (10 mg/mL in purified water), 4.78 mL purified water and 50 μL of Novozymes experimental enzyme mixture NS-22201 was added, the tubes closed and placed into an Stuart Orbital Incubator (S1500) at 50°C and 250 rpm. Saccharification samples were obtained by filtering 1 mL of the saccharification mixture through a PTFE syringe filter. Samples were run on a Shimadzu HPLC with an AMINEX HPX-97P column (Bio rad, 300 x 7.8 mm) with purified water as mobile phase (0.6 mL/min). The column temperature was 85°C and acquisition was run for 20 min. Calibration standards with concentrations of 0.1, 1, 2 and 4 mg/mL of glucose, xylose, mannose, arabinose and galactose and 8 mg/mL of glucose were used. Glucose yields were calculated relative to the total glucan content of untreated biomass.

### Feedstock and Pulp Characterization

#### Moisture Content

Moisture content determination of both native biomass and recovered pulp was determined according to published procedures (Gschwend et al., 2018).

#### Compositional Analysis

Compositional analysis was carried out in triplicates on native biomass and air-dried pulps, following the NREL protocol ‘Determination of Structural Carbohydrates and Lignin in Biomass' (Sluiter et al., 2008). Details of the procedure used in our laboratory may be found in the Supplementary Material.

#### Delignification and Hemicellulose Removal

The pulp delignification was calculated using the following equation:

(1) $$\begin{array}{r} \text{Delignification}=\frac{{Lignin\;}_{untreated}-\left(Lignin_{pulp\times }Yield_{pulp}\right)}{{Lignin\;}_{untreated}}\cdot 100\% \end{array}$$

where *Lignin*_*untreated*_ is the total lignin content in untreated biomass, *Lignin*_*pulp*_ is the total lignin content in the pulp and *Yield*_*pulp*_is the oven-dried pulp yield.

Hemicellulose removal can similarly be obtained from Equation 2, shown below:

(2) $$\text{Hemicellulose removal​}=\text{​}\frac{Hem_{untreated}-(Hem_{pulp}\times Yield_{pulp})}{Hem_{untreated}}\cdot 100\%$$

Where *Hem*_*untreated*_ is the hemicellulose sugar content of the untreated biomass and *Hem*_*pulp*_ is the hemicellulose content of the pulp.

#### Ash Content Determination

Ash samples were obtained from all four feedstocks (~0.5 g original sample weight) by ashing at 575°C in air to remove any organic material using a ramping program in a muffle furnace (Nabertherm + controller P 330). The heating program was as follows: heating from room temperature to 105°C; hold at 105°C for 12 min to remove moisture; heating to 250°C at 10°C/min; hold at 250°C for 30 min; heating to 575°C at 10°C/min; hold at 575°C for 3 h; cool to 105°C. Ash contents were determined in triplicate.

#### Elemental Analysis

CHNS analysis of untreated air-dried biomass was performed in duplicate by MEDAC Ltd. (Chobham, UK) by dynamic flash combustion analysis and thermal conductivity detection. Oxygen content was obtained by difference. Accuracy is ±0.30% absolute.

### Lignin Analysis

#### Enzymatic Mild Acidolysis Lignin (EMAL)

Enzymatic mild acidolysis lignin (EMAL) was isolated from depithed sugarcane bagasse based on a three-stage protocol adapted from Wu and Argyropoulos (2003). First, depithed sugarcane bagasse was ground to < 0.75 mm using a cutting mill. It was then acetone-extracted for 48 h and dried in a vacuum oven at 45°C. Approximately 60 g of dry extracted biomass was subjected to planetary ball milling (Retsch PM400, Germany) for 14 days at a rotation frequency of 150 rpm. It was ground in four 250 mL ball mill jars in the presence of four stainless steel balls which occupied ~50% of the active jar volume, using toluene as a grinding aid. The biomass was then allowed to dry in a fume cupboard overnight until the toluene had evaporated. Following ball-milling, enzymatic mild acid hydrolysis was performed in two separate stages. In the first stage, the ball-milled biomass was treated with cellulase enzymes (Ctec2, Novozymes, Denmark) in the amount of 190 mg protein per g biomass. The enzymatic hydrolysis was carried out at 50°C for 48 h at 3% consistency in the presence of 2% Tween 20 in 0.1 M citrate buffer (pH ~4.75). The slurry was stirred in a 2 L jacketed borosilicate glass pressure vessel (polyclave, Büchiglasuster, Switzerland) that was agitated using an anchor impeller at 120 rpm. The process temperature was controlled using an oil recirculator equipped with a thermostat (Unistat 405, Huber, Germany). After 48 h had elapsed, the slurry was centrifuged at 3,000 rpm for 15 min with a high-speed centrifuge (Megastar 3.0, VWR, UK). The insoluble materials were re-suspended in a fresh batch of enzyme/citrate mixture for another 48 h at 50°C under the same reaction conditions. The insoluble materials were again collected by centrifugation and washed twice with acidified (pH 2.0) deionized water to remove soluble sugars. Residual proteins on the surface of the insoluble solids were washed twice with 6 M guanidine hydrochloride followed by freeze-drying for 72 h to obtain a crude lignin sample. In the second stage, the crude lignin was finely ground in a pestle and mortar and subjected to mild acid hydrolysis using an azeotrope of dioxane-water (96:4 v/v) containing 0.01 M HCl at 87°C under a nitrogen atmosphere. Butyl hydroxytoluene (BHT) was added as a radical scavenger and stabilizer to limit the formation of explosive peroxides. The crude lignin was suspended in the dioxane-water azeotrope with a solid-to-liquid ratio of 1:20 (g/mL) and refluxed for 2 h. After the reaction, the suspension was centrifuged and the supernatant was carefully withdrawn. The solid residue was washed with fresh dioxane-water until the supernatant was clear. The combined supernatants were then neutralized with sodium bicarbonate and then added drop-wise into 8 L acidified water (pH 2.0). The precipitated lignin was allowed to equilibrate with the aqueous phase overnight and was recovered by centrifugation and washed twice with deionized water. The solid was then washed once with hexane to dissolve any extractives and residual BHT and the purified solid was freeze-dried. The final yield of EMAL recovered through the entire procedure was 3 wt% based on dry bagasse starting material, and that based on the acid-insoluble lignin content of bagasse was 15 wt%.

#### HSQC NMR Spectroscopy of Lignins

^13^C-^1^H heteronuclear single quantum coherence (HSQC) NMR spectroscopy was performed in monoplicate for bagasse EMAL and for lignin precipitates generated under the optimized conditions for each of the following feedstocks: sugarcane bagasse (170°C, 45 min); rice husk (170°C, 45 min); rice straw (170°C, 30 min); wheat straw (170°C, 30 min). The analysis followed the same procedure described by Brandt-Talbot et al. (2017). The signal intensities were normalized to the total abundance of G_2_ + G_2,cond_ units. The total (G_2+_ G_2,cond_) and (S_2_ + S_2,cond_) were used to calculate the S/G ratio, after halving the contribution from S units to avoid double-counting for the symmetrical syringyl unit. The degree of condensation was estimated as the proportion of G_2_ units involved in condensation reactions, i.e. G_2,cond_ : (G_2+_ G_2,cond_).

## Results and Discussion

### Feedstock Characterization

In this study, four of the most abundant (>40 Mtpa) (FAOSTAT, 2018) lignocellulosic feedstocks available annually as crop waste were pretreated with a view to bioethanol production. The materials investigated were wheat straw, rice straw, rice husk and depithed sugarcane bagasse. For each material, the annual availability and geography, current uses and challenges for utilization, and appearance before pretreatment are presented in Table 1. All feedstocks were prepared by washing, air-drying and cutting to a standard size fraction (0.18–0.85 mm). Prior to pretreatment, the elemental and chemical composition of the feedstocks was determined (see Table S1 in the Supplementary Material).

**Table 1:** Agricultural residues investigated in the present study and their appearance after milling and before pretreatment.

**Feedstock**	**Annual availability and major geography^a^**	**Current uses and challenges**	**Appearance**
Wheat straw	43 Mtpa; Europe	Animal bedding (60%). Inefficient burning leads to alarming levels of environmental pollution	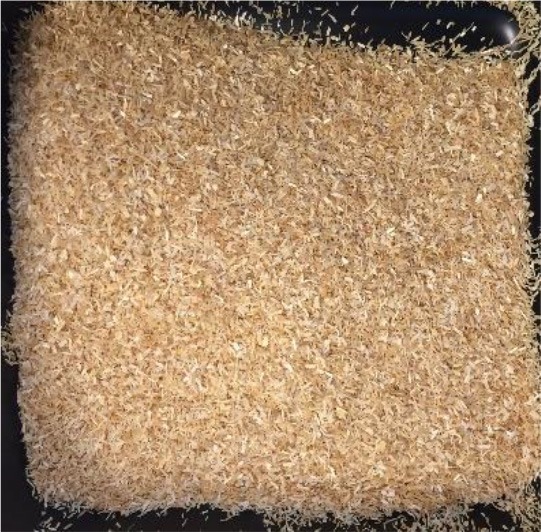
Rice straw	501 Mtpa; Asia, notably China and India	Burning in open fields (50%); fodder; landfilled. Logistical challenges for utilization as mostly left in fields. Inefficient burning leads to alarming levels of environmental pollution	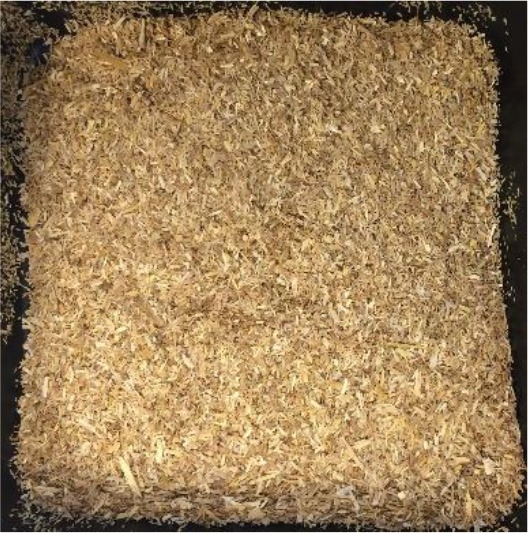
Rice husk	125 Mtpa; Asia, notably China and India	Combustion and gasification after forming pellets or briquettes (unknown %). Resistant to burning; abrasive to grinding equipment; poor nutritive value; high ash content	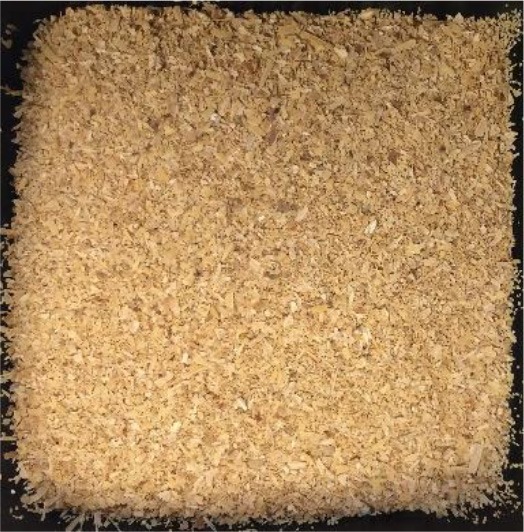
Sugarcane bagasse	157 Mtpa; Brazil, India and China	Combustion for combined heat and power production in sugar and ethanol mills (50%); remainder is stockpiled	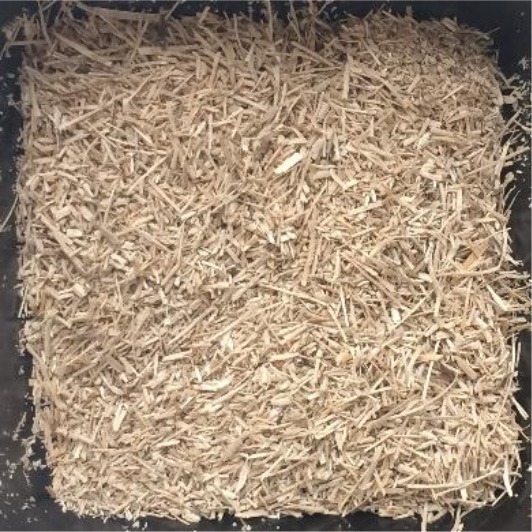

a *Based on 2017 data on crop production obtained from FAOSTAT (2018) and geographical availability from Satlewal et al. (2018). Key assumptions: bagasse to processed sugarcane ratio of 26:100 kg/kg (Li and Khanal, 2017); rice husk to rice paddy ratio of 22:100 kg/kg (Zafar, 2018); rice straw to paddy ratio of 400:100 kg/kg (IRRI, 2018); wheat straw to harvested wheat ratio of 43: 100 kg /kg available as straw (Agricultural Horticulure Development Board (AHDB), 2018); 40 wt% of available residues are plowed back into the soil for soil conservation (Scarlat et al., 2010)*.

The biomass had compositions typical of grassy feedstocks (Parikh et al., 2005; Ang et al., 2012), though significant variation was seen in their lignin and ash contents. Rice husk (27%) and sugarcane bagasse (24%) had the highest lignin content while wheat straw (22%) and rice straw (18%) were less lignin-rich. The most ash-rich feedstocks were rice straw and rice husk (13 and 11% total inorganic matter, respectively) as determined by heating to 575°C, a method which tends to give higher ash values than compositional analysis. Acid hydrolysis in the latter method measures only water- and acid-insoluble inorganic species as metals, whereas inorganic matter from ashing of whole biomass is present as oxides. The straws showed greater discrepancies between their acid-insoluble ash and total ash contents, possibly because of higher alkali species content which are acid-soluble (Miles et al., 1996). Elemental composition of the biomass materials showed that feedstocks with higher ash contents had correspondingly lower carbon contents, another factor in the low energy densities of rice residues (Mehta and Pitt, 1976).

All materials had similar cellulose and hemicellulose contents (70–72 wt%) making them suitable for conversion into bioethanol; the exception is rice husk, which had a lower polysaccharide content of 62% on account of its high ash and lignin contents.

### Fractionation Effectiveness for Rice Husk

**Figure 1:**
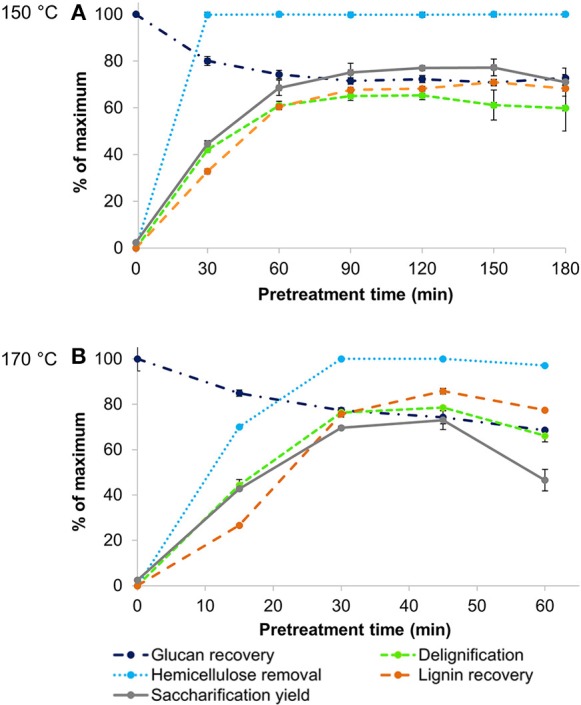
Key indicators of pretreatment effectiveness for Ionosolv pretreatment of rice husk at **(A)** 150°C and **(B)** 170°C with [TEA][HSO_4_], a water content of 20 wt% and a biomass to solvent ratio of 1:10 g/g.

Rice husk is naturally rich in lignin and has a significant ash content, which makes it the most recalcitrant and interesting feedstock to be studied. A first set of experiments was conducted by treating rice husk with the protic IL [TEA][HSO_4_] at two different temperatures (150° and 170°C) for pretreatment times between 30 min and 180 min. These time courses enabled the identification of optimal pretreatment conditions, assessed via the maximum saccharification yield from wet pulps (reported as the glucose released relative to the glucan content in native biomass). Figure 1 shows a number of pretreatment outcomes monitored over time for each temperature: lignin and hemicellulose removal from biomass, glucan retention in the pulp, and the yield of lignin precipitate as well as glucose yield after 7 days of enzymatic saccharification.

Pulp saccharification was the main indicator used to gauge pretreatment effectiveness. A number of studies have suggested that hornification, induced by air-drying, directly causes an irreversible pore shrinkage and collapse of pores within the cell wall, reducing internal surface area and, in turn, limiting enzyme accessibility and thereby lowering sugar yields (Luo et al., 2011). In this study, enzymatic saccharification was carried out for pretreated pulps both with and without an air-drying step. The outcomes (shown in Figure S1) confirmed that omitting an air-drying step for the pulp significantly increased the glucose yields by an average factor of 1.7. Very similar findings were obtained by Gschwend et al. (2019), whereby sugar release increased by 1.6 × for pine treated under comparable conditions. Consequently, *wet* pulps were directly subjected to saccharification in the rest of the study. This is in line with industrial practice, where the cellulose pulp would be subjected to enzymatic hydrolysis immediately after its recovery as a wet solid, rather than undergoing an energy-intensive air-drying step. For both time courses, two different peaks in saccharification yields were observed at the following conditions: after 2 h at 150°C and only 45 min at 170°C, where glucose yields reached 77 and 73%, respectively.

Compositional analysis of the pulps confirmed that hemicellulose and lignin were rapidly removed from the pulp by the acidic IL at both temperatures, while glucan remaining in the pulp degraded slowly over time. At both temperatures, hemicellulose was completely extracted by the IL after 30 min of treatment. A strong relationship was observed between pulp delignification and saccharification yields (Figure 1). After the point of peak saccharification and delignification, the pulp lignin was seen to increase again. We attribute this to lignin re-deposition onto the pulp surface, limiting the accessibility of the cellulose and exerting a negative effect on pulp digestibility, as studied in detail in our previous work (Brandt-Talbot et al., 2017; Weigand et al., 2017; Gschwend et al., 2018). For lignin recoveries to reach a maximum, a longer pretreatment time was required, which is related both to condensation of small lignin fragments dissolved in the ionic liquid yielding water-insoluble molecules that precipitate, and to pseudo-lignin formation (Gschwend et al., 2018). The formation of pseudo-lignin is expected at conditions when lignin recoveries exceed lignin removal, leading to a decrease in pulp accessibility and reduced sugar yields.

For the purpose of developing an industrially relevant process, the selected optimum condition for rice husk pretreatment was 45 min at 170°C, as shorter pretreatment times are desirable to reduce reactor volumes and hence capital cost (Gschwend et al., 2018).

### Fractionation of Four Agricultural Residues

After observing the strong performance of ionoSolv fractionation for the recalcitrant material rice husk, similar pretreatments were conducted at 170°C to optimize fractionation conditions for three other abundant agricultural residues, namely rice straw, wheat straw and sugarcane bagasse. Optimal pretreatment conditions were again selected on the basis of peak cellulose digestibility (rather than hemicelluloses), as cellulose is the most abundant component with higher value and large and mature market.

As Table 2 shows, the optimum glucose yields obtained from saccharification of wet pulps of all four feedstocks were very promising, as they are approaching the theoretical maximum glucose release based on the glucan content of untreated biomass. While 73% glucose yield was seen for rice husk, glucose release of nearly 90% was obtained for rice straw, wheat straw and sugarcane bagasse. We compared these yields to applications of the aprotic ionic liquid [Emim][OAc] for pretreatment of rice straw and rice husk, which gave maximal glucose yields of 75 and 40%, respectively (Ang et al., 2012; Poornejad et al., 2014). These findings show that [TEA][HSO_4_] outperforms cellulose-dissolving ILs at 1/40th of the solvent cost (George et al., 2015).

**Table 2:** Summary of maximal glucose yields obtained after Ionosolv pretreatment of for agroresidues at 170°C using [TEA][HSO_4_], a water content of 20 wt% and a biomass to solvent ratio of 1:10 g/g.

**Feedstock**	**Optimal pretreatment condition**	**Saccharification yield^a^**		**Increase in saccharification yield^b^**
		**With air drying (%)**	**Without air drying (%)**	
Wheat straw	170°C, 30 min	80.5 ± 0.8	86.5 ± 1.9	5.6
Rice straw	170°C, 30 min	68.3 ± 0.5	88.8 ± 0.2	3.4
Rice husk	170°C, 45 min	58.8 ± 1.5	73.0 ± 4.3	30.4
Sugarcane bagasse	170°C, 45 min	78.4 ± 2.1	89.4 ± 5.5	8.1

a *Calculated as a percentage of glucan content in untreated biomass*.

b *Calculated as ratio of wet pulp sugar yield compared to sugar yield for untreated biomass*.

Wheat straw (87%) and rice straw (89%) gave high glucose yields after only 30 min of treatment. We attribute this to their less dense and more porous cell wall structure as well as lower lignin content, meaning that fractionation is easier and less energy-intensive. Eighty-nine percent glucose recovery was obtained from sugarcane bagasse after 45 min of treatment, significantly higher than our previous optimum of 65% glucose yield from air-dried pulps at 120°C, presumably due to process intensification and direct saccharification of wet pulps reducing the effects of hornification. Rice husk gave 73% glucose yield, a 30-fold improvement over the very low glucose yield for untreated rice husk, which demonstrates that this material is highly recalcitrant (see Table S2 in the Supplementary Material). The fact that rice husk and bagasse required more prolonged treatment (45 min rather than 30 min) is consistent with their higher lignin contents (see Table S1), and their dense and closed cell wall structures ((Real et al., 1996; Pu et al., 2013); Satlewal et al., 2018).

**Figure 2:**
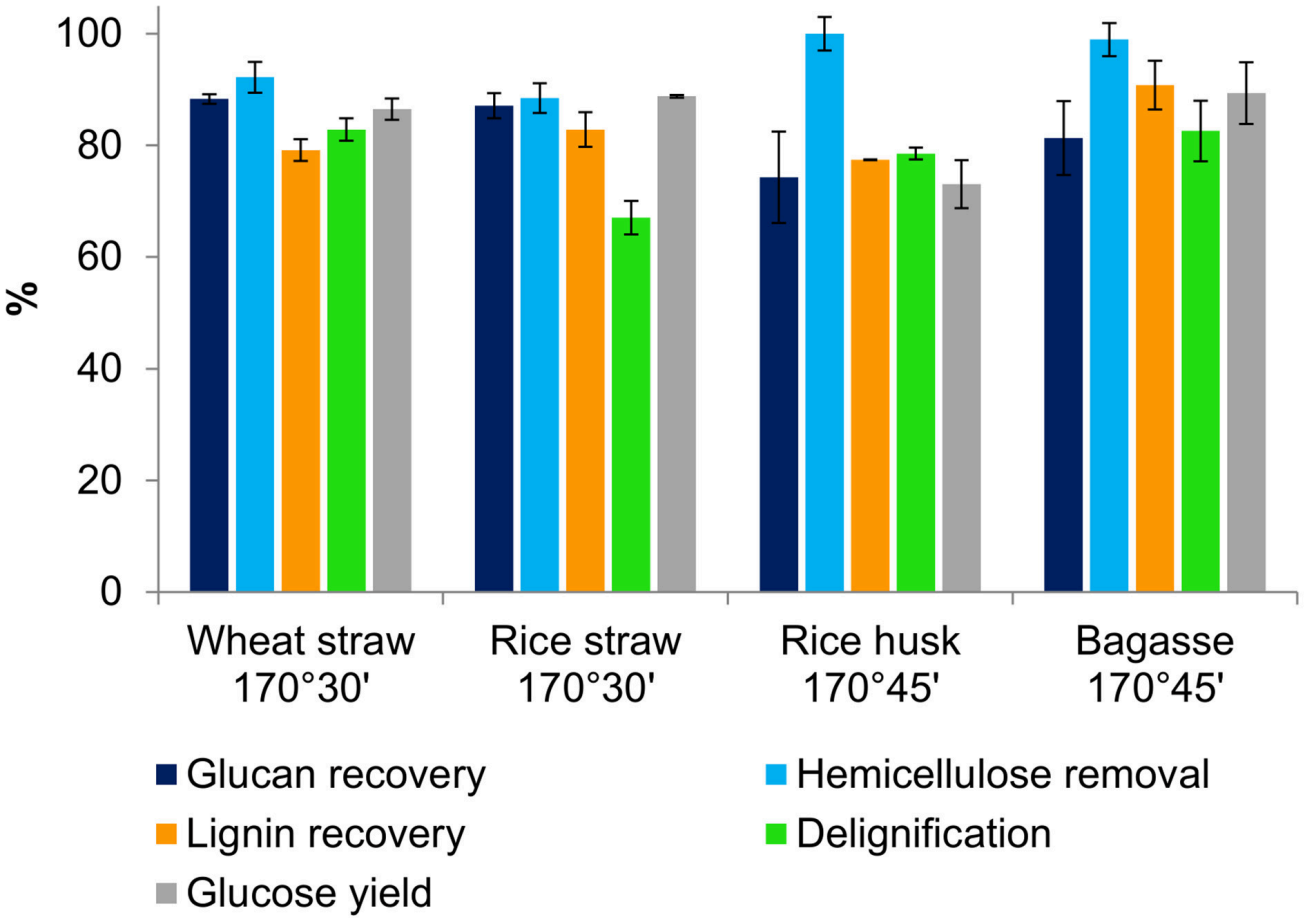
Comparison of key pretreatment parameters for agricultural residues following Ionosolv pretreatment with [TEA][HSO_4_] under optimized conditions.

Figure 2 presents the key compositional changes of all four feedstocks under the optimal conditions for glucose hydrolysis identified in Table 2. Under these conditions, the hemicelluloses can be seen to have nearly completely hydrolysed into the IL solution. The hemicellulose solutes are known to react further in the acidic IL to form degradation products such as furfural and acetic acid, which can be separated quantitatively from the non-volatile IL by distillation (Brandt-Talbot et al., 2017). More prolonged treatments led to nearly quantitative hemicellulose removal, but this was accompanied by 10–20% glucan loss, as glucan degrades rapidly at 170°C. Cellulose degradation, rather than delignification, is suspected to be the limiting factor for glucose release. This was investigated by comparing the glucose yields as a percentage of glucan in the *pulp* (rather than in untreated biomass), shown in Table S2 in the Supplementary Material. This analysis showed that glucan that survived the treatment was quantitatively released, suggesting that further optimization may be possible by adjusting reaction times between 30 and 45 min to maximize glucan recovery and hence glucose release from enzymatic saccharification. Lignin was also dissolved from the pulp by the acidic IL and recovered as a precipitate by addition of water to the IL liquor. All four feedstocks produced pulps that were highly delignified (67–82%), reflecting the strong link between lignin removal and glucose release; quantitative lignin removal is rarely observed (Brandt-Talbot et al., 2017). High lignin precipitate yields of ~80–90% were obtained, with values similar to lignin removal from the pulp for all feedstocks, except for rice straw and bagasse where the lignin recovery slightly exceeded delignification. The latter, which seems to indicate the “formation” of lignin, strongly suggests the occurrence of condensation reactions occurring between lignin fragments; upon further treatment, lignins form insoluble macromolecules that condense onto the pulp, reducing glucose yields, as seen in our previous work (Brandt-Talbot et al., 2017; Gschwend et al., 2018).

### Lignin Characterization

**Figure 3:**
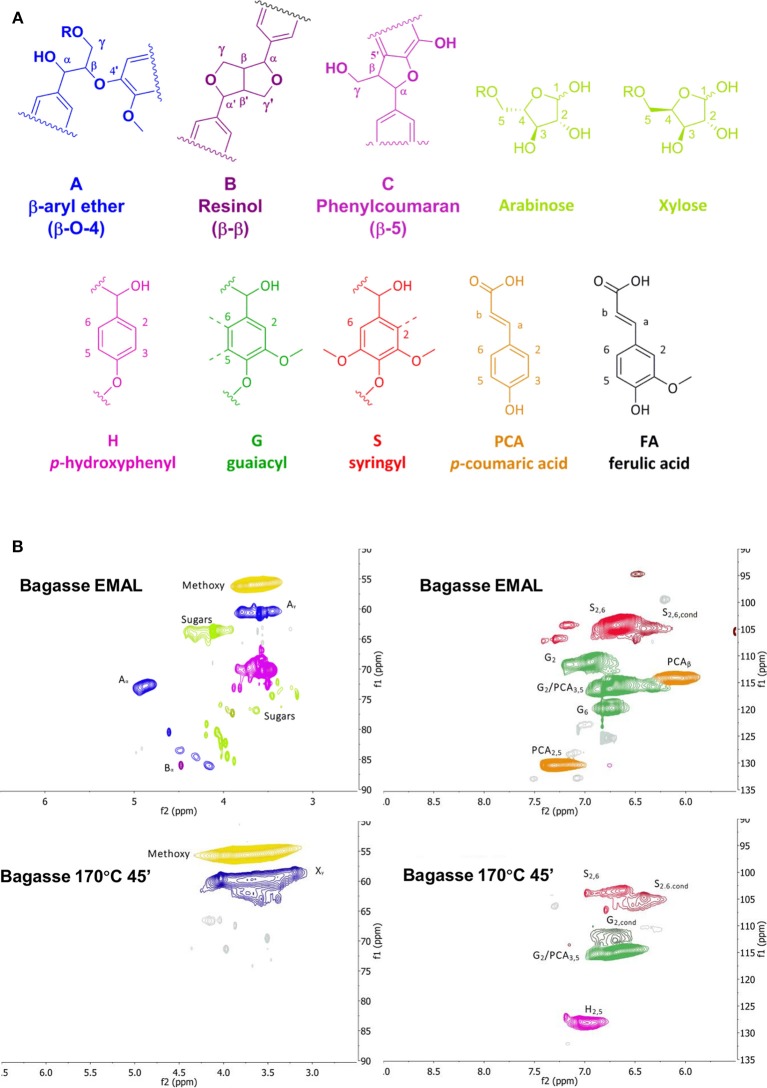
**(A)** Lignin substructures found in grassy lignins, and **(B)** HSQC NMR spectra of bagasse enzymatic mild acidolysis lignin and lignin isolated with [TEA][HSO_4_], with a water content of 20 wt% and a biomass to solvent ratio of 1:10 g/g. Side chain region (left) and aromatic region (right).

As was shown in the previous section, pulp delignification is closely linked to the overall pretreatment performance represented by the saccharification yield. Compositional analysis does not provide information about changes in lignin structure and inter-unit linkages during pretreatment, which is key information for value-added applications of lignin. Therefore, we set out to analyze the lignin fractions isolated under the optimized pretreatment conditions at 170°C using HSQC NMR spectroscopy. Enzymatic mild acidolysis lignin (EMAL) was used to represent native lignin and as a benchmark against which the isolated ionoSolv lignins can be compared. Figure 3A shows the main lignin structural units present, while the two key regions of the NMR spectra for bagasse EMAL and bagasse ionoSolv lignin can be seen in Figure 3B. Full spectra of lignins isolated from all four agricultural feedstocks are available in Figure S2 (see Supplementary Material).

**Figure 4:**
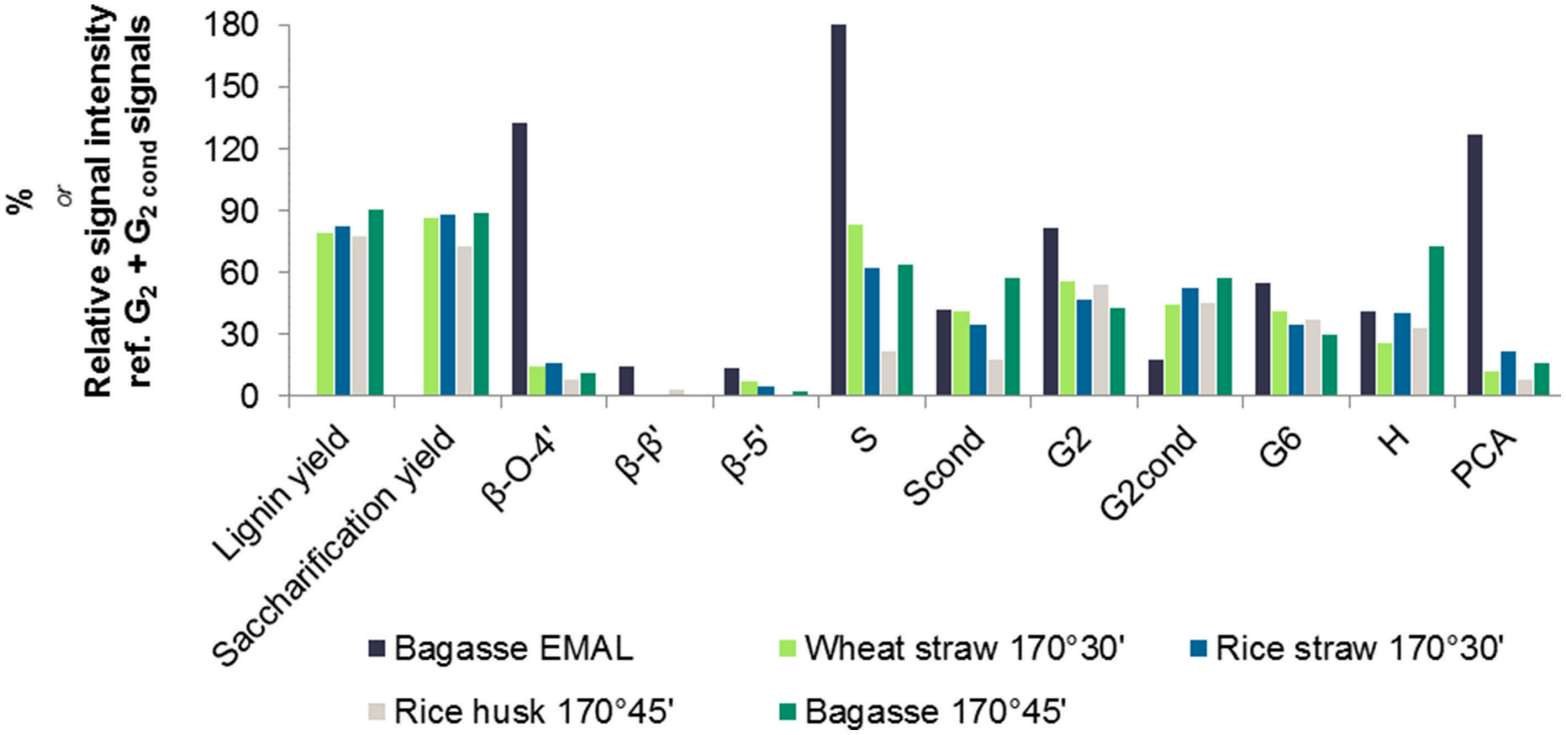
HSQC NMR signal intensities of different lignin C-H units relative to 100 G_2_ + G_2,cond_ units, obtained for enzymatic mild acidolysis lignin (EMAL) isolated from sugarcane bagasse and for lignins isolated from four agricultural feedstocks with [TEA][HSO_4_] with 20 wt% water and a biomass to solvent ratio of 1:10 g/g.

A semi-quantitative technique was applied to quantify the changes in lignin sub-units following ionoSolv pretreatment. The results of volume integration are presented in Figure 4. All signal intensities were compared to the sum of the G_2_ and G_2,cond_ integrals, which can be used as an internal standard in the case of herbaceous biomass, according to the findings of Brandt-Talbot et al. (2017). It is thought to be the most stable C-H subunit in lignin and therefore representative of the total number of aromatic units in lignin (Zhang and Gellerstedt, 2007). Signal intensities were compared in Figure 4 and the S/G ratios and degree of condensation of G_2_ units are presented in Table 3.Within the side chain region of the spectrum (δ*H* 6.5 – 2.5; δ*C* 90 – 50) seen in Figure 3B, the most obvious change is the disappearance of signals corresponding to sugars from the pretreated lignin spectrum, reflecting high lignin purity. Delignification is known to proceed through cleavage of α-*O*-4′ and β-*O*-4′, linkages, facilitating dissolution of lignin into the IL (Brandt et al., 2015). The signals assigned to the major interunit linkages β-*O*-4′, β-5′, and β-β′ linkages disappeared after pretreatment, as they are rapidly cleaved or modified in acidic media. Among the three bonds, β-*O*-4′ ether linkages were the most readily removed during the acidic pretreatment, with over 90% of bagasse ether bonds gone after pretreatment (Figure 4). Decreased signal intensities assigned to β-5′ and β-β′ linkages were attributed to their chemical alteration in acidic media rather than cleavage, as these linkages contain C–C bonds which are unlikely to have been broken (Brandt et al., 2015).

**Table 3:** Degree of condensation as evidenced by S/G ratio and condensed: non-condensed G_2_ ratios of enzymatic mild acidolysis lignin (EMAL) isolated from sugarcane bagasse and for lignins isolated from four agricultural feedstocks with [TEA][HSO_4_] with 20 wt% water and a biomass to solvent ratio of 1:10 g/g.

**Lignin**	**Sub-unit composition**				**S/G ratio^a^**	**Cond/Non-cond^b^**
	**G_**2**_**	**G_**2,cond**_**	**S_**2**_**	**S_**2,cond**_**		
Bagasse EMAL	81.8	18.2	182.5	42.5	1.12	0.18
Wheat straw 170°30′	55.8	44.2	83.3	41.3	0.62	0.44
Rice straw 170°30′	47.1	52.9	62.0	34.9	0.48	0.53
Rice husk 170°45′	54.5	45.5	22.2	17.5	0.20	0.45
Bagasse 170°45′	42.9	57.1	64.3	57.9	0.61	0.57

a *Calculated from HSQC NMR spectra signals as 0.5(S_2_ + S_2,cond_)/(G_2_ + G_2,cond_)*.

b *Calculated as cond/non-cond = G_2,cond_/(G_2_ + G_2,cond_)*.

Within the aromatic region (δ*H* 9.0 – 5.5; δ*C* 135 – 90), extensive condensation between aromatic rings was observed. Condensation between aromatic rings is thought to occur at the 2- and 6- ring positions of G and S lignin units which are not fully substituted (Brandt et al., 2015). From Figure 4, evidence for C–C condensation reactions in bagasse lignin can be seen in the decrease in G_2_ and S and increase in G_2,cond_ and S_cond_ signals, relative to the sum of G_2_ and G_2,cond_ signals. Compared to native bagasse lignin (i.e. EMAL), the signal intensity of G_6_ units was also reduced, which is similarly thought to stem from C–C condensation reactions occurring at this position. The degree of condensation of G_2_ units, seen in Table 3, showed that around 50% of G_2_ units had undergone condensation reactions in all pretreated feedstocks, compared to only 18% in bagasse EMAL. Rice straw and bagasse lignin appeared to be comparatively more condensed, as seen in their higher G_2,cond_ : G_2_ ratios (Figure 4; Table 3), in agreement with the evidence for more advanced lignin condensation seen from compositional analysis (Figure 2). Another distinctive feature is the much lower S/G ratio observed for rice husk lignin, which could be a factor in its recalcitrance (Pu et al., 2013). Additionally, a significant drop in *p*-coumaric acid (PCA) subunit content and an apparent increase in H subunits were observed within this NMR region. This is in agreement with our previous findings, whereby interdependence of PCA and H units was explained by conversion of PCA units into “H-like” IL-soluble oligomers under acidic solutions, with these reactions occurring in parallel with condensation reactions (Brandt et al., 2015; Brandt-Talbot et al., 2017). Given that extensive condensation may preclude several value-added applications of lignin, such as carbon fiber or chemicals production (Ragauskas et al., 2014), further fine-tuning of the process conditions may be necessary to simultaneously optimize lignin properties and pulp digestibility, as demonstrated by Gschwend et al. (2018).

### Fate of Ash Components

**Figure 5:**
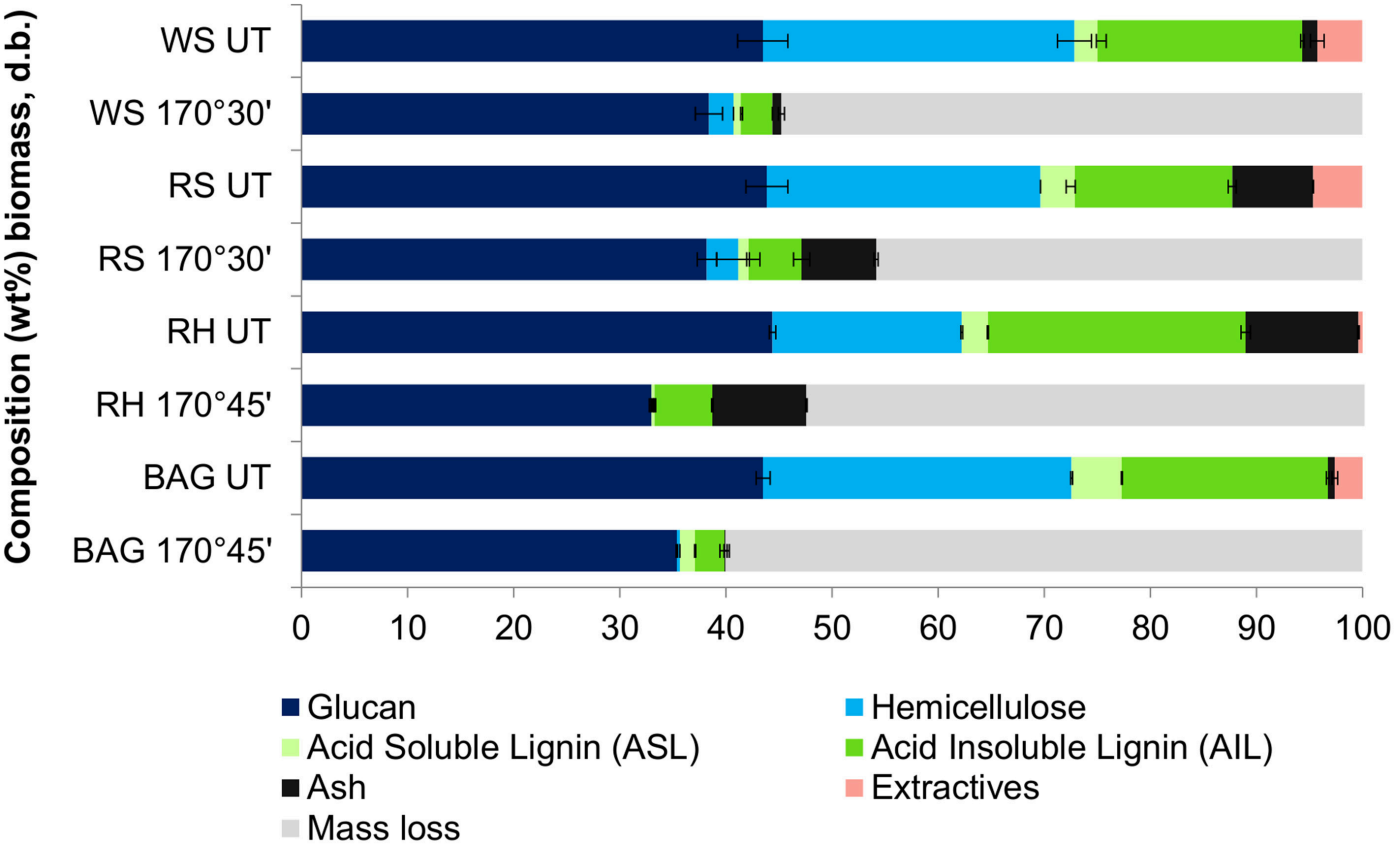
Chemical composition of untreated (UT) and pre-treated cellulose-rich pulps isolated from pretreatment of rice husk, sugarcane bagasse, wheat straw, and rice straw with [TEA] and [HSO_4_] under optimized conditions.

Ash recovery also needs to be considered when optimizing the pretreatment conditions, especially for rice straw and rice husk, which contain 13 and 11 wt% ash, respectively. Compositional analysis of the native biomass and pretreated pulps (Figure 5) revealed that ash mostly partitioned with the pulp, as expected from our previous study (Brandt-Talbot et al., 2017). This was most clearly observed for high-ash rice husk and rice straw, where 92 ± 5% and 84 ± 8% of ash were recovered in the pulp. It should be noted that chemical analysis reveals only acid-insoluble inorganic matter, whereas acid-soluble ash components are likely to have dissolved in the acidic ionic liquid medium during pretreatment (or later in the aqueous sulfuric acid medium during compositional analysis). The presence of ash in the pulp does not appear to have negatively impacted its enzymatic digestibility: between 88 and 100 wt% of the cellulose-rich material was hydrolyzed to glucose (see Table S2). The exception is rice husk, which was 58% hydrolyzed, presumably due to its greater residual lignin content and more packed cell wall structure (Real et al., 1996; Pu et al., 2013; Satlewal et al., 2018). As nearly all of the pulp can be hydrolyzed, the amount of post-hydrolysis solids would be desirably low, simplifying processing. The inorganic matter, especially silica, could also be easily recovered in the post-hydrolysis solids. Facile separation of ash from the post-hydrolysis solids is helpful as the ash often has market value; for instance, rice husk ash is silica-rich and has various applications in cement production and waste-water treatment (Foo and Hameed, 2009). The effect of an additional ash product stream on process economics remains to be investigated.

## Conclusions

Four of the world's most abundant agricultural residues, namely rice straw, rice husk, sugarcane bagasse and wheat straw, were pretreated using the ionoSolv process. The pretreatment solvent was the low-cost protic ionic liquid [TEA][HSO_4_] with a water content of 20 wt% and a biomass to solvent ratio of 1:10 g/g. It was applied to four agricultural feedstocks with high lignin (up to 28 wt%) and ash content (up to 13 wt%), which were successfully pretreated to yield a highly digestible cellulose-rich pulp and lignin as co-products. Optimum pretreatment conditions were identified at a temperature of 170°C with the aim of maximizing enzymatic saccharification yields and minimizing residence time. Rice straw and wheat straw could be pretreated within 30 min while more prolonged treatment of 45 min was needed for rice husk and sugarcane bagasse, which have more dense and lignified structures. The glucose yields obtained following enzymatic saccharification of the pretreated pulps were 73% for rice husk and close to 90% glucose yields for rice straw, wheat straw and sugarcane bagasse. Glucose release from the pulps was enhanced by a factor of ~1.7 by saccharifying wet pulps without an air-drying step to reduce hornification. Around 80–90% of lignin present in the feedstock could be recovered as a precipitate while the ash mostly partitioned within the solid pulp, enabling its recovery in the post-hydrolysis solids. According to both wet and dry pulp saccharification assays, [TEA][HSO_4_] was demonstrated to obtain significantly higher glucose yields than aqueous [Emim][OAc] in the pretreatment of agricultural residues at the laboratory scale (process volumes of 10 mL), despite having a solvent cost of 1/40th that of [Emim][OAc].

A strong link between residual pulp lignin and saccharification yields was noted. Delignification was seen to proceed *via* extensive cleavage of β-*O*-4′ aryl ether linkages which was accompanied by condensation reactions in the lignin precipitate. This work has validated the potential use of the ionoSolv fractionation technology for conversion of abundant agricultural residues to bioethanol. Further work in this area should establish the energy requirements of grinding silica-rich materials such as rice husk and straw before pretreatment, and finding value-added applications for the inorganic components. Moreover, the process needs to be intensified to further reduce solvent and energy consumption, notably by use of larger particle sizes, higher solids loadings and direct pretreatment of moist raw feedstocks after harvesting to eliminate the need for energy-intensive drying of feedstocks prior to pretreatment.

## Author Contributions

MC conducted all the major experiments and helped with the manuscript preparation. CC designed the study and wrote the manuscript. JH and PF provided valuable inputs for the study's development and helped with manuscript writing. All authors listed have made a substantial, direct and intellectual contribution to the work, and approved it for publication.

### Conflict of Interest Statement

The authors declare that the research was conducted in the absence of any commercial or financial relationships that could be construed as a potential conflict of interest.
