# Supplementary material for: Efficient Fractionation of Lignin- and Ash-Rich Agricultural Residues Following Treatment With a Low-Cost Protic Ionic Liquid
Source: Front Chem. 2019 Apr 17;7:246. doi: 10.3389/fchem.2019.00246 (PMC6478884; doi:10.3389/fchem.2019.00246)
Supplement: Supplementary file 1 [file Table_1.DOCX]

Supplementary Material

Efficient Fractionation of Lignin- and Ash-rich Agricultural Residues Following Treatment with a Low-Cost Protic Ionic Liquid

Clementine L. Chambon^1^, Meng Chen^1^, Paul S. Fennell^1^, Jason P. Hallett^1*^

*** Correspondence:** Prof Jason P. Hallett : [j.hallett@imperial.ac.uk](mailto:j.hallett@imperial.ac.uk)

# Ionic liquid synthesis

The ionic liquid triethylammonium hydrogen sulfate ([TEA][HSO_4_]) was synthesized according to a previously published protocol (Gschwend et al., 2018). Trimethylamine was cooled in an ice bath to which 5 M sulfuric acid was added dropwise. Water was removed using a rotary evaporator until the water content in the ionic liquid solution was 20 wt%. A Karl-Fisher titrator (V20 volumetric titrator, Mettler-Toledo, USA) was used to verify the ionic liquid moisture content according to the standard operating procedure of our laboratory (Gschwend et al., 2016). ^1^H NMR spectra were conducted in a Bruker 400 MHz spectrometer to verify IL purity.

^1^H NMR: δH (400 MHz, DMSO-d_6_)/ppm: 3.39 (s (br), *H*SO_4_^-^, N-*H*^+^), 3.10 (q, J = 7.3 Hz, 6H, N-C*H*_2_), 1.20 (t, J=7.3 Hz, 9H, N-CH_2_-C*H*_3_). ^13^C NMR: δC (101 MHz, DMSO-d^6^)/ppm: 46.21 (N-C*H*_2_), 9.15 (N-CH_2_-C*H*_3_).

# Compositional analysis

300 mg (calculated on ODW basis) of air-dry biomass or recovered biomass was weighed out into a pressure tube and the weight recorded. 3 mL of 72% sulfuric acid was added, the samples stirred with a Teflon stir rod and the pressure tubes placed into a preheated water bath at 30°C. The samples were stirred again every 15 min for one hour, then diluted with 84 mL distilled water and the lids closed. The samples were autoclaved (Sanyo Labo Autoclave ML5 3020 U) for 1 hour at 121°C and left to cool to close to ambient temperature. The samples were then filtered through filtering ceramic crucibles of a known weight. The filtrate was filled in two Falcon tubes and the remaining black solid washed with distilled water. The crucibles were placed into a convection oven (VWR Venti-Line 115) at 105°C for 24±2 hours. They were then taken out and placed in a desiccator for 15 min before they were weighed and the weight recorded. They were then placed into a muffle oven (Nabertherm + controller P 330) and ashed to constant weight at 575°C. The weight after ashing was recorded. The content of acid insoluble lignin (AIL) was determined according to Equation S1. The content of one of the Falcon tubes was used for the determination of acid soluble lignin content (ASL) by UV analysis at 240 nm (Equation S2) (Perkin Elmer Lambda 650 UV/Vis spectrometer).

$\%AIL=\frac{{Weight}_{crucible plus AIR}-{Weight}_{crucible plus ash}}{{ODW}_{sample}}\cdot100$ (Eq. S1)

$\%ASL=\frac{A}{l\cdot\varepsilon\cdot c}\cdot100=\frac{A\cdot V_{filtrate}}{l\cdot\varepsilon\cdot{ODW}_{sample}}\cdot100$ (Eq. S2)

where *Weight_crucibles plus AIR_* is the weight of the oven-dried crucibles plus the acid insoluble residue, *Weight_crucibles plus ash_* is the weight of the crucibles after ashing to constant temperature at 575°C, A is the absorbance at 240 nm, l is the pathlength of the cuvette in cm (1 cm in this case), ε is the extinction coefficient (12 L/g cm for agricultural feedstocks), c is the concentration in mg/mL, ODW is the oven-dried weight of the sample in mg and V_filtrate_ is the volume of the filtrate in mL and equal to 86.73 mL. The AIL and ASL contents were combined and reported as the total lignin content.

The glucan content and hemicellulose content of the pulp can be derived from Equation S3.

$\text{G}\text{lucan}\text{ or hemicellulose }\text{content }\text{=}\frac{C_{HPLC}\times V\times{corr}_{anhydro}}{SRC\times{ODW}_{sample}}\cdot100$ (Eq. S3)

where c*_HPLC_* represents the sugar concentration detected by HPLC, *V* is the initial volume of the sugar solution (10.00 mL for the sugar recovery standards and 86.73 mL for all sugar samples), *corr_anhydro_* is a correction factor for the mass increase during polymeric sugar hydrolysis (0.90 for glucose, galactose and mannose, 0.88 for xylose and arabinose) and $SRC$ stands for sugar recovery coefficient(0.949 for glucose, 0.878 for galactose, mannose, xylose and arabinose).

Calcium carbonate was added to the contents of the second falcon tube until the pH reached 5. The liquid was passed through a 0.2 µm PTFE syringe filter and subsequently submitted to HPLC analysis (Shimadzu, Aminex HPX-97P from Bio rad, 300 x 7.8 mm, purified water as mobile phase at 0.6 ml/min, column temperature 85°C) for the determination of total sugar content. Calibration standards with concentrations of 0.1, 1, 2 and 4 mg/mL of glucose, xylose, mannose, arabinose and galactose were used. Sugar recovery standards were made as 10 mL aqueous solutions close to the expected sugar concentration of the samples and transferred to pressure tubes. 278 µL 72% sulfuric acid was added, the pressure tube closed and autoclaved and the sugar content determined as described above. The sugar recovery coefficient (SRC) was determined according to Equation S4 and the sugar content of the analysed sample using Equation S5:

$SRC=\frac{c_{HPLC}\cdot V}{initial weight}$ (Eq. S4)

$\%Sugar=\frac{c_{HPLC}\cdot V{\cdot corr}_{anhydro}}{SRC\cdot{ODW}_{sample}}\cdot100$ (Eq. S5)

where c_HPLC_ is the sugar concentration detected by HPLC, V is the initial volume of the solution in mL (10.00 mL for the sugar recovery standards and 86.73 mL for the samples), initial weight is the mass of the sugars weighed in, corr_anhydro_ is the correction for the mass increase during hydrolysis of polymeric sugars (0.90 for C6 sugars glucose, galactose and mannose and 0.88 for C5 sugars xylose and arabinose) and ODW is the oven-dried weight of the sample in mg.

# Feedstock characterisation

Table S1. Elemental and chemical composition of agroresidues investigated in this study, as determined by CHNS analysis and compositional analysis (reported on a wt% dry basis).

|  |  | Wheat straw | Rice straw | Rice husk | Sugarcane bagasse |
| --- | --- | --- | --- | --- | --- |
| Elemental composition | C | 48.0 | 41.8 | 41.2 | 48.3 |
|  | H | 5.2 | 4.9 | 4.7 | 5.3 |
|  | N | 0.8 | 1.1 | 0.8 | 0 |
|  | S | <0.1 | <0.1 | 0.5 | 0.7 |
|  | O ^a^ | 46.1 | 52.2 | 52.8 | 45.7 |
| Compositional analysis | Cellulose | 43.5 | 43.9 | 44.4 | 43.5 |
|  | Hemicellulose | 29.4 | 25.8 | 17.8 | 29.1 |
|  | Lignin | 21.5 | 18.1 | 26.8 | 24.1 |
|  | Extractives | 4.3 | 4.6 | 0.4 | 2.6 |
|  | Acid-insoluble ash | 1.4 | 7.6 | 10.6 | 0.7 |
|  | Total ash at 575 °C | 3.7 | 12.7 | 10.7 | 0.7 |

^a^ Calculated by difference (O = 100 – C – H – N – S, wt %).

# Effect of pulp drying on saccharification yields

Figure S1. Glucose yields for air-dried (dashed) and wet (continuous) pulps for rice husk pretreated at 150 °C and 170 °C. Inset values show factor of increase in glucose yield for wet : dry pulps.

# Saccharification of four agricultural feedstocks

Table S2. Glucose release after 7 day enzymatic saccharification experiments for a range of pretreated samples and untreated biomass. Data is expressed as four different percentages of glucose release.

|  | **Glucose release** | | | |
| --- | --- | --- | --- | --- |
|  | **As % of untreated biomass** | **As % of glucan in untreated biomass** | **As % of pretreated pulp** | **As % of glucan in pretreated pulp** |
| Wheat straw untreated | 6.7±0.1 | 15.4±0.2 | - | - |
| Wheat straw  170 °C 30 min | 37.6±1.0 | 86.5±1.9 | 90.4±3.7 | 98±0.2 |
| Rice straw untreated | 11.4±3.4 | 26.0±6.3 | - | - |
| Rice straw  170 °C 30 min | 38.8±0.1 | 88.2±0.2 | 88.0±5.3 | 102.0±0.2 |
| Rice husk untreated | 1.08±0.02 | 2.4±0.0 | - | - |
| Rice husk  170 °C 45 min | 31.0±1.4 | 73.0±4.2 | 57.5±2.4 | 98.2±0.2 |
| Bagasse untreated | 4.8±0.1 | 11.4±2.8 | - | - |
| Bagasse  170 °C 45 min | 38.9±2.9 | 89.4±5.5 | 100.1±5.6 | 109.8±5.6 |

# Lignin HSQC NMR spectra


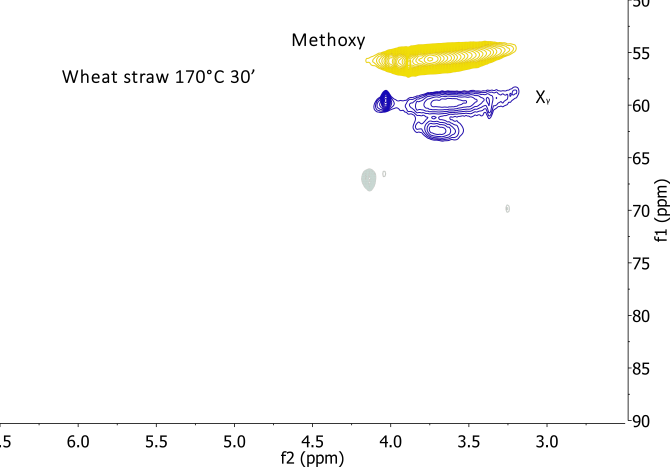

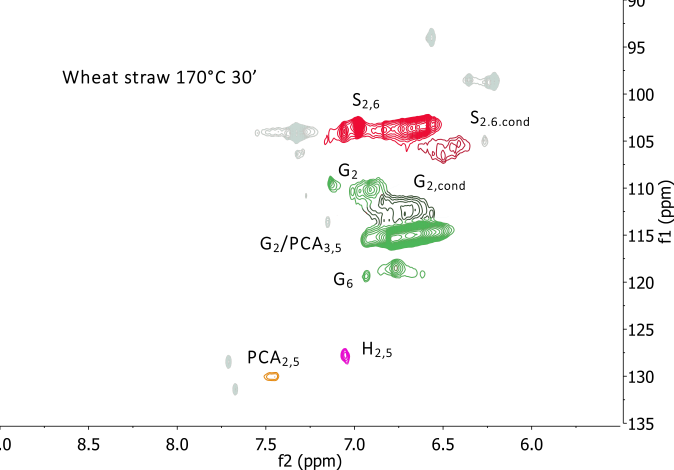


**Wheat straw 170°C 30’**

**Wheat straw 170°C 30’**


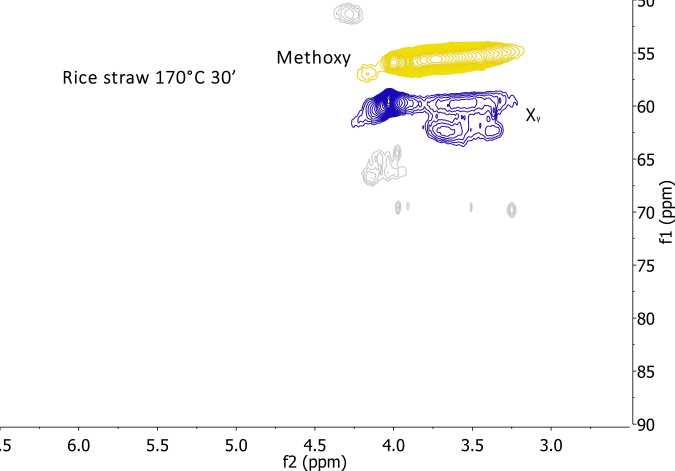

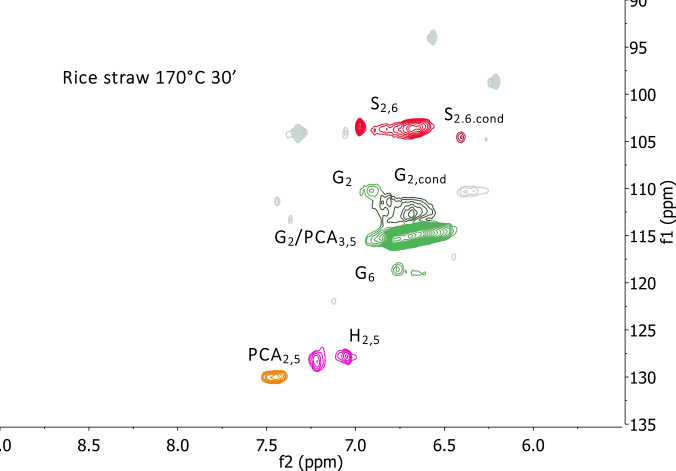


**Rice straw 170°C 30’**

**Rice straw 170°C 30’**


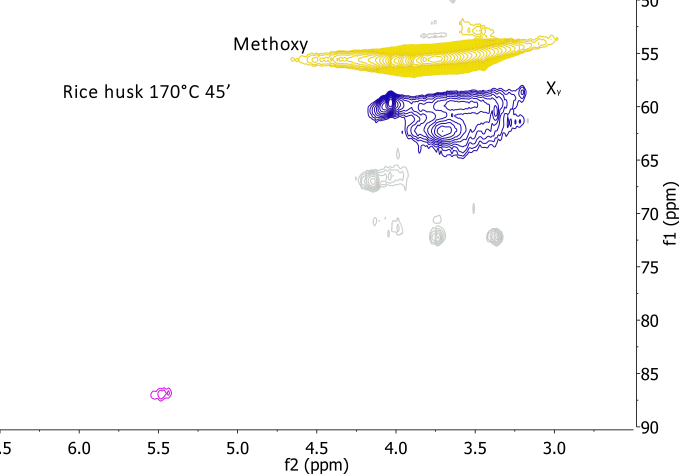

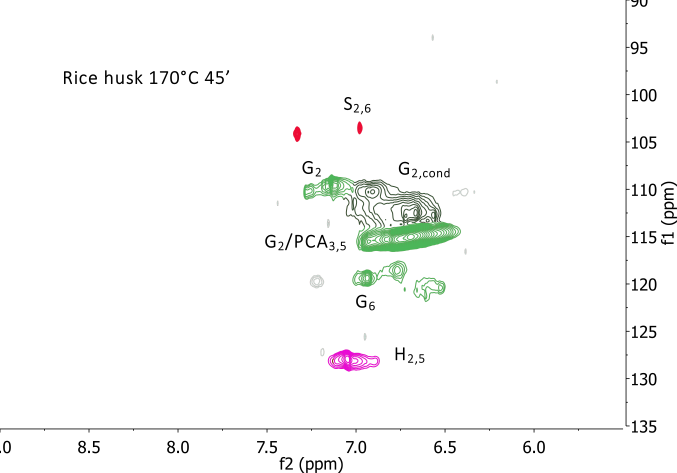


**Rice husk 170°C 45’**

**Rice husk 170°C 45’**


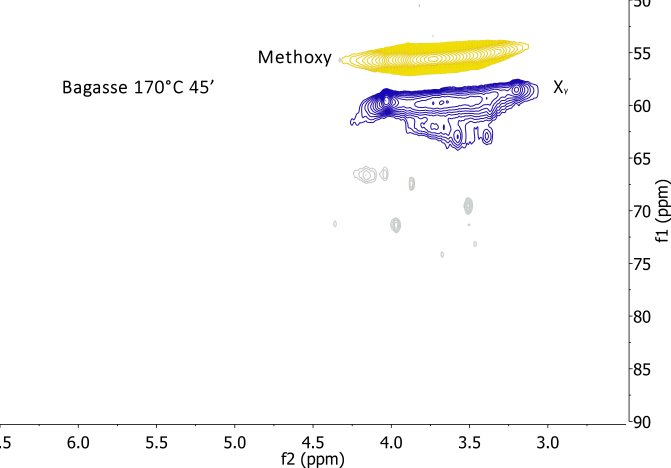

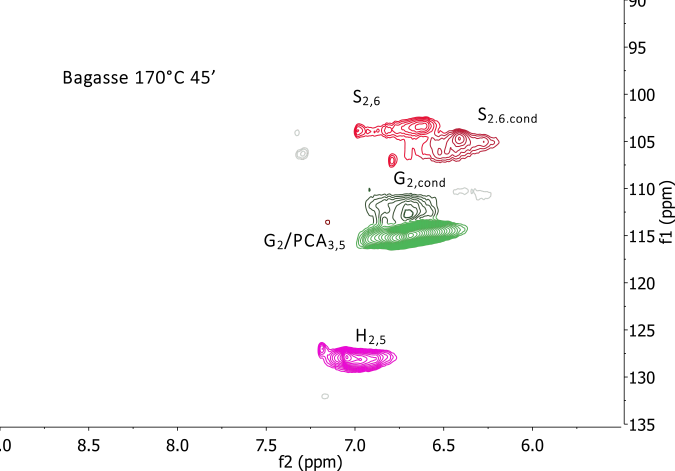

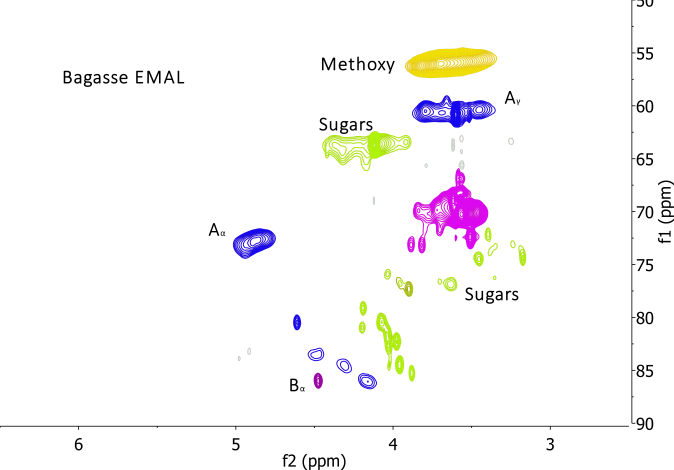

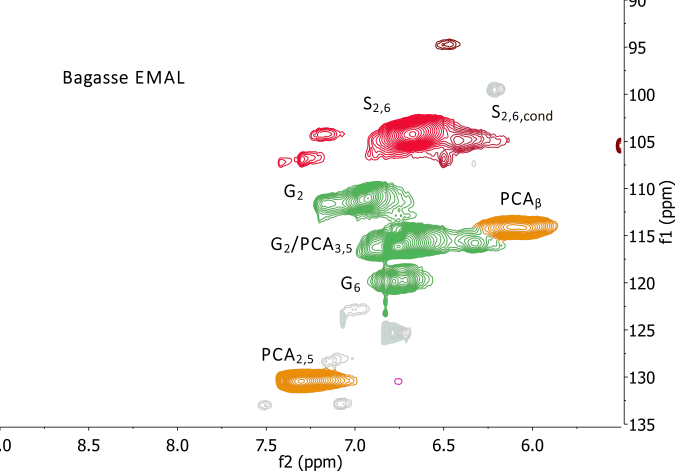


**Bagasse EMAL**

**Bagasse EMAL**

**Bagasse 170°C 45’**

**Bagasse 170°C 45’**

Figure S2. HSQC NMR spectra of lignins isolated from wheat straw, rice straw, rice husk and sugarcane bagasse after extraction with [TEA][HSO_4_], with a water content of 20 wt% and a biomass to solvent ratio of 1 : 10 g/g, and enzymatic mild acidolysis lignin (EMAL) extracted from bagasse. Side chain region (left) and aromatic region (right).
